# Experimental and theoretical approaches for the selective detection of thymine in real samples using gold nanoparticles as a biochemical sensor†

**DOI:** 10.1039/c8ra02627k

**Published:** 2018-07-05

**Authors:** Kamlesh Shrivas, Nidhi Nirmalkar, Santosh Singh Thakur, Ramsingh Kurrey, Deepak Sinha, Ravi Shankar

**Affiliations:** School of Studies in Chemistry, Pt. Ravishankar Shukla University Raipur CG-492010 India kshrivas@gmail.com +91-7752-260488; Department of Chemistry, Guru Ghasidas Vishwavidyalaya Koni Bilaspur CG India; Department of Chemistry, Government Nagarjuna Post Graduate College of Science Raipur CG-492010 India; Nanoscience and Nanoengineering Program, South Dakota School of Mines and Technology Rapid City South Dakota-57701 USA

## Abstract

We report a simple, selective and cost effective method for the qualitative and quantitative determination of thymine in a DNA standard and urine samples using gold nanoparticles (AuNPs) as a label-free colorimetric biochemical sensor. The mechanism for the detection of thymine is demonstrated *via* the color change of the AuNPs from pink to blue, followed by the shift of the localized surface plasmon resonance (LSPR) absorption band to a higher wavelength with the introduction of an analyte. The selective detection of thymine was experimentally verified by performing a control experiment with nucleobases, other biomolecules, metal ions and anions. In addition, the computation density functional theory (DFT) and time dependent density functional theory (TD-DFT) using the Gaussian (C.01) program highlighted that the electrostatic potential behavior of the thymine molecule facilitated a non-covalent interaction toward gold for the selective detection of analytes, and the computation was also used to calculate a UV-Vis absorption band as well. The calculated absorption band of the AuNPs with thymine, obtained using TD-DFT, was found to be very close to the experimental data. The omnicapped truncated tetrahedral (*ν*_3_-tetrahedral) Au_20_ cluster structure was considered as the model for the AuNP optimization. The linear range obtained for the quantitative determination of thymine was found to be 10–1200 ng mL^−1^ with a limit of detection of 3 ng mL^−1^. The advantages of using the AuNPs as a biochemical sensor are that they provide a facile and low cost method and are selective for the qualitative and quantitative determination of thymine in a DNA standard and in urine samples in comparison to chromatographic and electrochemical methods.

## Abstract 2

We report a simple, selective and cost effective method for the qualitative and quantitative determination of thymine in a DNA standard and in urine samples using gold nanoparticles (AuNPs) as a label-free colorimetric biochemical sensor.

## Graphical abstract

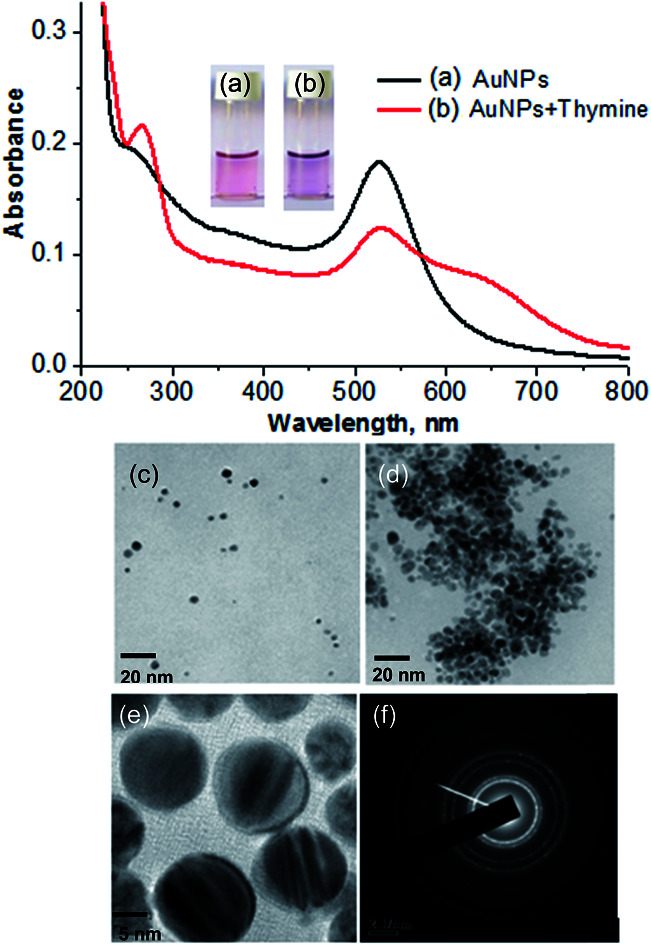

## Introduction

Deoxyribonucleic acid (DNA) is an important biomolecule that is used in living organisms to carry genetic information from one generation to another. DNA is made up of two strands of polynucleotide containing a phosphate group and a sugar group, with nitrogen bases such as adenine, thymine, guanine and cytosine.^1,2^ Abnormal changes to thymine or a deficiency of thymine in DNA may result in mutation or an irregularity in the immune system and may result in symptoms of mental retardation, cancer, ageing, cardiovascular disease, renal failure and other diseases.^3^ Therefore, the qualitative or quantitative determination of thymine in biological samples has a great significance for clinical diagnosis.

Various analytical techniques like gas chromatography (GC),^4^ high performance liquid chromatography (HPLC),^5^ nuclear magnetic resonance (NMR) spectrometry,^6^ cyclic voltammetry (CV),^7,8^ fluorescence,^9^ the immunocytochemical method (ICM)^10^ and colorimetry^11^ are used for the determination of thymine in a variety of samples. GC, HPLC, NMR, CV and the ICM method are found to be expensive and tedious with laborious sample preparation steps prior to instrumental analysis. The colorimetric method is simple, economic, rapid and can be applied at the sample source due to the small size of the instrument. The conventional colorimetric method has the disadvantage of employing a specific chromophoric reagent to form a colored complex with the target analyte. Occasionally, the chromophoric reagent is not selective because it reacts with other materials in the sample matrix. In addition, there are few methods available for the quantitative determination of thymine from biological samples. Therefore, a simple colorimetric method is required that should be free from a chromophoric reagent (label-free) and selective for detecting thymine in complex sample matrices.

Recently, the use of nanomaterials in the field of analytical science has provided a cost effective, efficient and eco-friendly option for rapid and sensitive detection of a variety of analytes from different types of sample. Nanomaterials have the ability to capture or bind a target analyte selectively, which makes them predominantly attractive to use as chemical or biochemical sensors.^12–14^ Noble metal nanoparticles (NPs) such as gold (Au), silver (Ag) and copper (Cu) are widely used as colorimetric sensors for the detection of a variety of analytes because of their optical and electronic properties such as their localized surface plasmon resonance (LSPR). LSPR is an oscillation of free electrons present on the conduction band of metal NPs, which is induced *via* interaction with the visible light section of an electromagnetic wave and which has the characteristic of an LSPR absorption band in the visible region. This property of the metal NPs makes them excellent colorimetric sensors for the determination of a variety of analytes in environmental, biological, food, clinical and pharmaceutical samples.^15–18^ Recently, interest in colorimetric sensors has expanded because of their potential to detect target analytes in sample solutions. In this context, we have demonstrated the use of AgNPs and AuNPs for the colorimetric sensing of pesticides,^19,20^ cationic surfactants,^21^ metal ions^22–24^ and drugs^25^ from biological, environmental and food samples. In addition, the interaction of noble metal NPs with oligonucleotides and their building substances such as nucleobases and nucleosides has been reported in the literature.^26,27^ Zheng *et al.* demonstrated the use of surfactant capped AgNPs as a resonance light scattering probe for the determination of nucleic acid through the electrostatic and chemical affinity forces between NPs and analyte molecules.^28^ Zhou *et al.* demonstrated the colloid stability of thymine-functionalized AuNPs and elucidated the shift in the plasmon band of NPs with a change in surface charge, particle size and solvent type. The end group of the thymine molecule strongly interacted with the surface of the AuNPs in a self-assembled manner to form normal alkanethiols.^29^ Therefore, we attempted to develop an analytical method for the determination of thymine in sample solutions using AuNPs as a biochemical sensor.

To the best of our knowledge, there is no literature available on the selective detection of thymine in the presence of other nucleobases using AuNPs as a label-free biochemical sensor based on the electrostatic potential behavior of the analyte towards the gold. The high surface-to-volume ratio of the AuNPs is found to be good for the interaction of the AuNPs with thymine molecules. We have developed a colorimetry-based biochemical sensor by exploiting the optical properties of the AuNPs (such as LSPR) in the visible light region for qualitative and quantitative determination of thymine in a DNA standard and in urine samples. The linearity, limit of detection, precision and accuracy of the newly developed method were evaluated for the determination of thymine in urine samples.

## Experimental section

### Reagents and solution preparations

All the chemicals used were of analytical grade. Auric chloride (HAuCl_4_), sodium dodecylbenzene sulfonate (SDBS), haemoglobin, guanine, thymine, adenine, cytosine, bilirubin, glucose, urea and sodium borohydride (NaBH_4_) were purchased from Himedia (Mumbai, India). The structures of all of the nucleobases along with glucose, urea and bilirubin are shown in the ESI in Fig. S1.† The standard stock solutions of the biomolecules were prepared by dissolving an appropriate amount of each substance in 10 mL of distilled water (DW) or methanol. The working standard solutions were prepared by diluting the stock standard solution. 0.1 M NaOH and 0.1 M HCl solutions were prepared and used for maintaining the pH of the sample solution.

### Synthesis of dodecylbenzene sulfonate (DBS)-capped AuNPs

A wet chemical method was employed for the synthesis of DBS-capped AuNPs *via* the reduction of AuHCl_4_ using NaBH_4_ as a reducing agent in the presence of DBS as a stabilizing agent. Briefly, 100 mL of 1.0 × 10^−3^ M HAuCl_4_ and 1 mL of 0.2 M SDBS were added into a 250 mL conical flask and the solution mixture was stirred for 30 min at room temperature. After this, 1 mL of 0.2 M chilled NaBH_4_ was added drop-wise and the color of the solution mixture changed from pale yellow to pink, showing the formation of DBS-capped AuNPs. The prepared AuNPs were stored in a refrigerator at 5 °C prior to the analysis of thymine *via* colorimetry.

### Sample preparation for the determination of thymine in a DNA standard and in urine samples

A known quantity of a DNA standard solution was added into a glass bottle containing 600 μL of formic acid and the sealed bottle was kept at 150 °C for 30 min. The obtained solution was neutralized with 0.1 M NaOH for the qualitative detection of thymine using the AuNPs as a biochemical sensor. The urine samples were collected in cleaned polyethylene bottles from healthy volunteers with the help of trained personnel. Consent was obtained from the people concerned for the performance of the experiment. All the experiments were performed in compliance with the relevant laws and institutional guidelines as well as with the approval of a research committee. The samples were filtered with Whatman filter paper no. 42. An aliquot of the urine sample (1 mL) was added into a glass vial and hydrolyzed with formic acid at 150 °C for 30 min, followed by neutralization with NaOH solution.^30^ The neutralized solution of urine was utilized for the quantitative determination of thymine using the AuNPs as a biochemical sensor.

### Procedure for the detection of thymine using the AuNPs as a biochemical sensor

An aliquot of the standard solution of thymine (1.0 mL) or pretreated DNA standard or urine sample was added into a 5 mL glass vial containing 1.0 mL of the AuNPs while maintaining the pH of the sample solution at 7.0. The solution mixture was allowed to sit for 5 min of reaction time at room temperature. The color of the solution mixture changed from pink to blue depending on the addition of analyte into the AuNP solution. The color intensity of the solution mixture was quantitatively monitored using UV-Vis spectrophotometry.

### Apparatus

The signal intensity and LSPR absorption peak in the determination of thymine were monitored using a UV-Vis spectrophotometer (Shimadzu, Tokyo, Japan) from 200–800 nm. The shape and size of the AuNPs were measured with a transmission electron microscope (TEM) at an accelerated voltage of 100 kV. TEM images of the AuNPs were obtained with and without the addition of thymine after placing 1 μL of a dilute aqueous solution of the NPs on the copper grid. The dynamic light scattering (DLS) measurements were performed using a Nano-Zetasizer instrument (Malvern, UK) to determine the distribution and hydrodynamic size of the AuNPs in aqueous solution before and after the addition of thymine.

## Results and discussion

### Characterization of the AuNPs

**Fig. 1:**
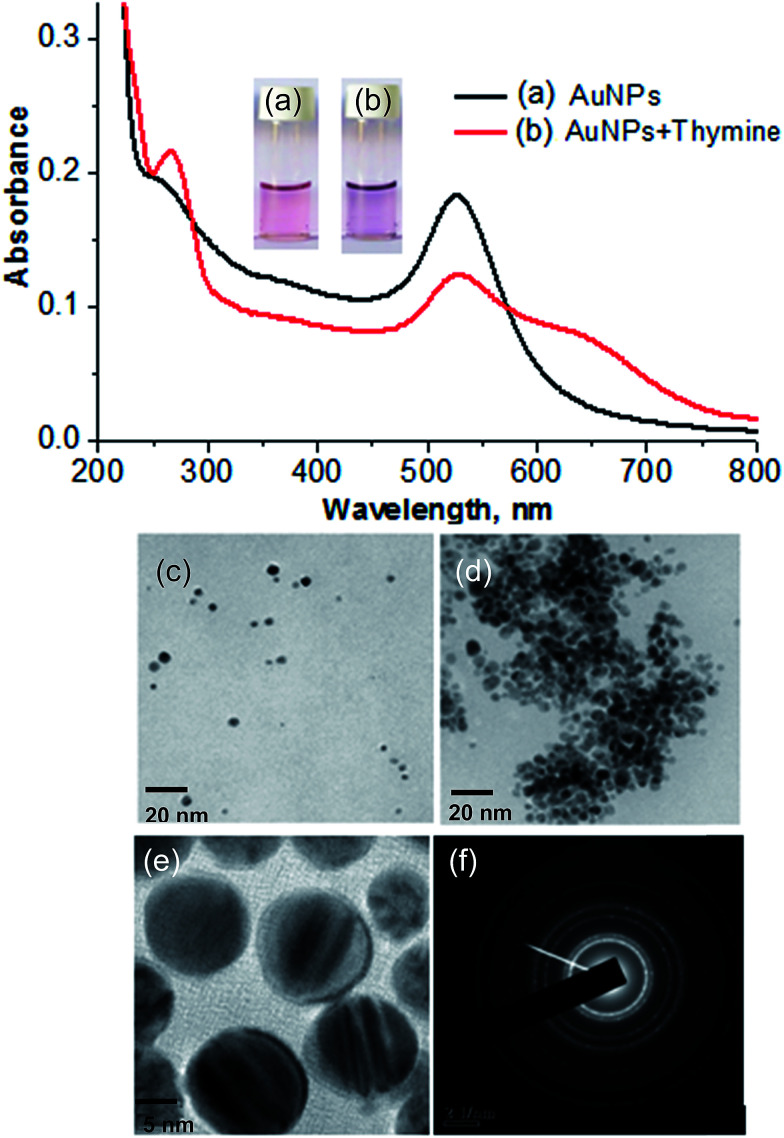
A glass vial containing (a) the AuNPs and (b) the AuNPs with thymine, along with their respective UV-Vis spectra. TEM images of (c) the AuNPs and (d) the AuNPs with thymine. (e) The enlarged view of the AuNPs with HR-TEM and (f) the lattice structure of the AuNPs.

**Fig. 2:**
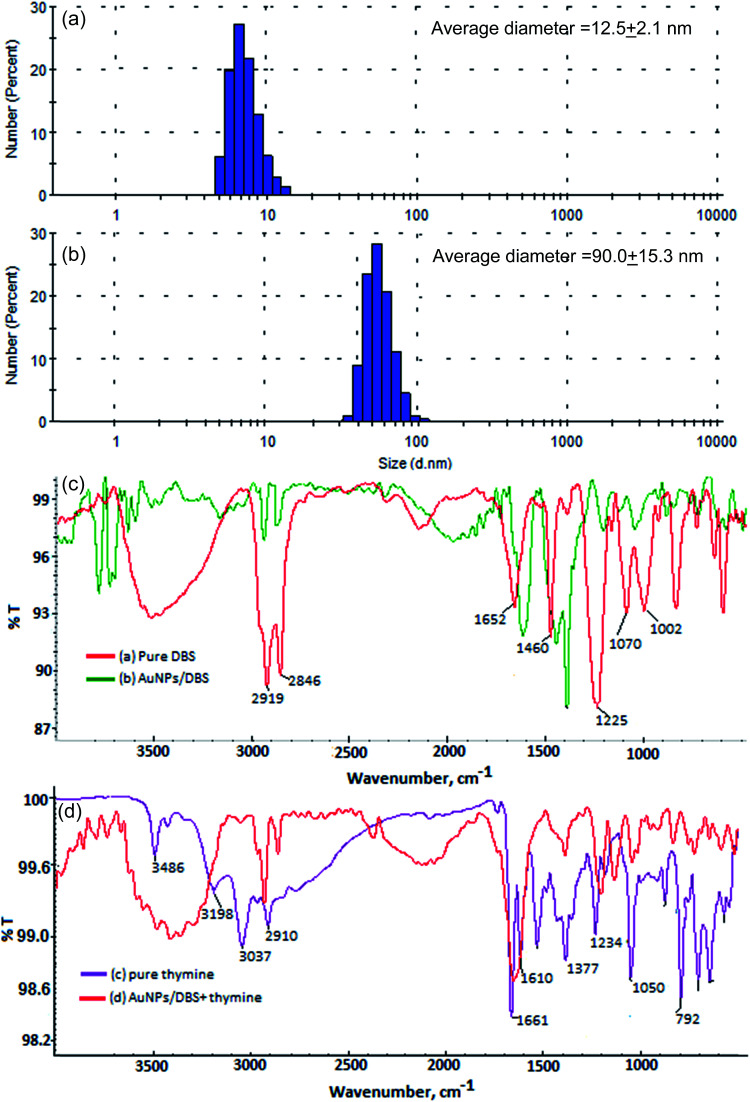
DLS measurements of (a) the AuNPs and (b) the AuNPs with thymine showing the percentage distribution of the particles before and after the addition of thymine. FTIR spectra of (c) pure DBS and AuNPs/DBS and (d) pure thymine and the AuNPs with thymine.

The formation of DBS-capped AuNPs was first noticed *via* a color change of the aqueous solution of HAuCl_4_ from yellow to pink after the addition of a reducing agent (NaBH_4_). The sharp LSPR absorption peak at 525 nm in the visible region showed the formation of stable DBS-capped AuNPs, as shown in Fig. 1(a). The reason for the red shift of the LSPR band was the aggregation of the DBS-capped AuNPs. The addition of thymine into the AuNPs caused the LSPR band to shift to a higher wavelength from 525 nm to 650 nm, as shown in Fig. 1(b). TEM measurements were carried out before and after the addition of thymine into the AuNPs to measure the size of the AuNPs. The AuNPs modified with DBS exhibited monodispersity in aqueous solution and the average size of the NPs was found to be 11.5 + 1.5 nm, as shown in Fig. 1(c). However, there was an increase in the size of the NPs after the introduction of thymine, as shown in Fig. 1(d). The enlarged view and different lattice structure of the AuNPs are shown in Fig. 1(e) and (f). DLS measurements were also performed to obtain the size distribution of the AuNPs in aqueous solution before and after the addition of thymine. The increase in size of the AuNPs was seen in the aggregated particles compared to the mono-disperse NPs, as shown in Fig. 2(a) and (b). The results acquired through the DLS measurements agreed well with the results of the TEM and UV-Vis measurements. The surface modification of the AuNPs with DBS was confirmed using FTIR analysis of pure DBS and the AuNPs capped with DBS, as shown in Fig. 2(c). Intense peaks were acquired at 2915 cm^−1^ and 2846, which were attributed to the asymmetric and symmetric stretching of the CH_2_ group of the aliphatic carbon chain. The peak at 1460 cm^−1^ showed the C=C stretching vibration of the aromatic carbon in DBS. The peak observed at 1225 cm^−1^ was because of the asymmetric stretching of the sulphonic acid (head group) of DBS. The decreases in signal intensity at 1225 cm^−1^, as well as the shift to 1200 cm^−1^ in the spectrum, verified the capping of DBS onto the surface of the NPs.^31^ Next, the IR spectra of pure thymine and the solution mixture of the AuNPs with thymine were recorded. The results are shown in Fig. 2(d). The bands obtained at 3198 cm^−1^ and 1377 cm^−1^ corresponded to the NH stretching and bending vibrations, respectively. The peaks obtained at 3037 and 2910 cm^−1^ were due to the CH_3_ asymmetric and symmetric stretching of the thymine molecule. The peaks at 1661 and 1050 cm^−1^ were attributed to the C=O and C–O stretching vibrations.^32^ The decrease in the signal intensity and the shift of the bands were observed for the solution mixture of AuNPs + thymine compared to pure thymine molecules, demonstrating the interaction of thymine with the NPs.

### Assay for the selective detection of thymine using the AuNPs as a biochemical sensor

**Fig. 3:**
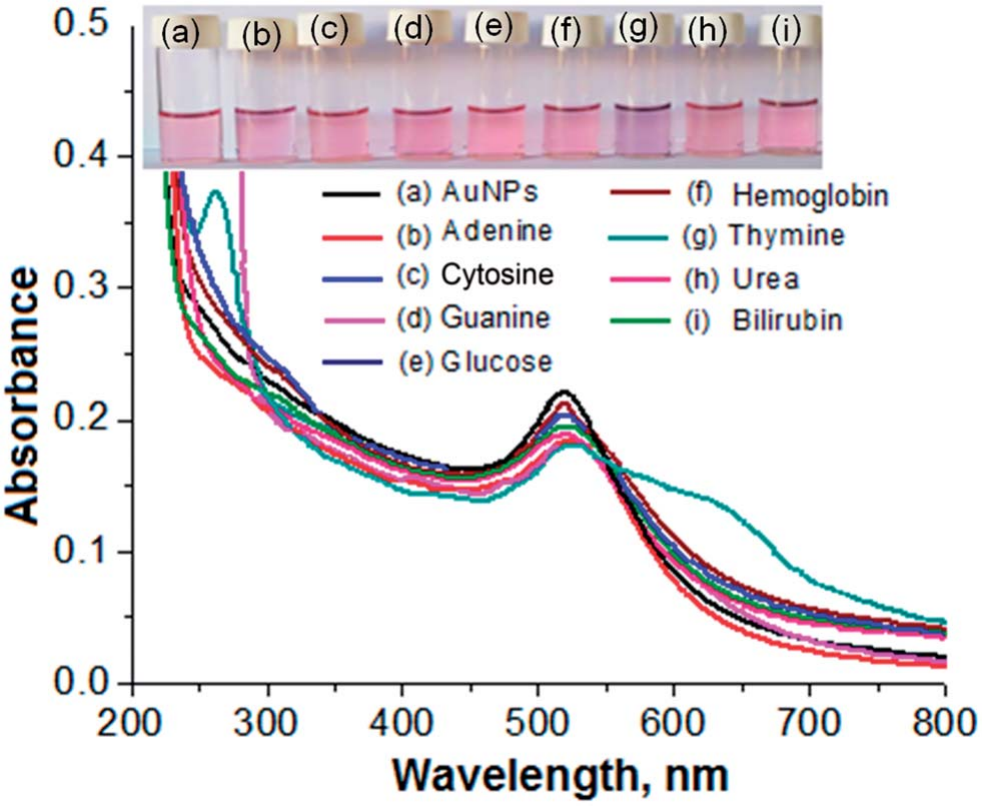
Images of glass vials containing the AuNPs with different biomolecules (500 ng mL^−1^): (a) the AuNPs, (b) adenine, (c) cytosine, (d) guanine, (e) glucose, (f) haemoglobin, (g) thymine, (h) urea and (i) bilirubin, along with their UV-Vis spectral data using the AuNPs as a biochemical sensor at pH 7.0 for 5 min of reaction time at room temperature.

Biomolecules such as adenine, cytosine, guanine, glucose, haemoglobin, thymine, urea and bilirubin were tested for selective detection of particular molecules from a sample solution using the AuNPs as a biochemical sensor. For this, an aliquot (1.0 mL) of each standard solution of the different biomolecules was spiked into a separate glass vial containing 1.0 mL of the AuNPs. Each of the solution mixtures was kept at room temperature for 5 min of reaction time. The results are shown in Fig. 3. The NP solution with thymine displayed a color change from pink to blue, as shown in Fig. 3(g). The solution mixtures with the other biomolecules did not exhibit the color change, demonstrating that there may be no interaction between these molecules and the NPs, as shown in Fig. 3(b)–(f), (h) and (i). In addition, the solution mixture of the AuNPs with thymine exhibited a red shift of the LSPR absorption band from 525 to 650 nm in the visible region. The solution mixtures of the AuNPs with the other biomolecules did not show any shift in the LSPR absorption band. Therefore, the color change from pink to blue and the red shift in the visible region after the addition of thymine into the AuNPs was selected for selective determination of the target analyte from a sample solution.

### Mechanism for the selective detection of thymine using the AuNPs as a biochemical sensor

The mechanism for the selective detection of thymine from a sample solution using the AuNPs as a biochemical sensor was investigated by performing different sets of experiments. The DBS-capped AuNPs were prepared by reducing gold salt (Au^3+^) with NaBH_4_*via* a simple wet chemical method. The DBS present on the surface of the AuNPs prevented the self-aggregation of colloidal particles due to the repulsive force of the negative charge of the capping agent acting against the van der Waals forces of the NPs. Thus, the AuNPs modified with sulfonate ions and long hydrophobic carbon chains in aqueous solution remain mono-disperse and exhibit a pink color with an LSPR absorption peak at around 525 nm in the visible region, as shown in Fig. 3(a). In contrast, the addition of thymine into the mono-dispersed NPs caused a color change from pink to blue, followed by the aggregation of the particles, which results in a red shift of the LSPR absorption band in the visible region from 525 nm to 650 nm (Fig. 3(g)). A theoretical TD-DFT calculation using the Polarizable Continuum Model (PCM) for the solvent water and the integral equation formalism variant (IEFPCM) using the self consistent reaction field method (SCRF) provided an absorption band at 654.8 nm (Fig S2 and Table S1 in ESI†), which is close to the experimental value. According to the classical theory of Derjaguin, Landau, Verwey and Overbeek (DLVO) for colloidal stability, the kinetic barrier of energy to aggregation comes from the collective effects of the van der Waals attraction and the electrostatic inter-particle repulsion.^33^ In neutral pH (7.0), a thymine molecule is uncharged and is able to replace the DBS from the surface of the AuNPs, which leads to a decrease in the surface charges of the NPs.^34^ This causes the rapid aggregation of the AuNPs in the presence of thymine in aqueous solution, followed by the color change and the shift of the LSPR band to a higher wavelength in the visible region.

Interestingly, the color change and the red shift of the LSPR absorption band were found only with the thymine molecule and not with other nucleobases such as adenine, guanine and cytosine. A literature survey suggested that nitrogen and oxygen atoms in the ring structure of the nucleobases have better binding sites. The structure of adenine and guanine are closely similar to each other with a pyrimidine ring with an amine group and an imidazole ring, which in guanine has an oxygen. Similarly, both thymine and cytosine have a pyrimidine ring in their structure and the difference between them is found at the 6-position in the pyrimidine ring, which contains an oxygen and amine group, respectively.^26,27,35^

**Fig. 4:**
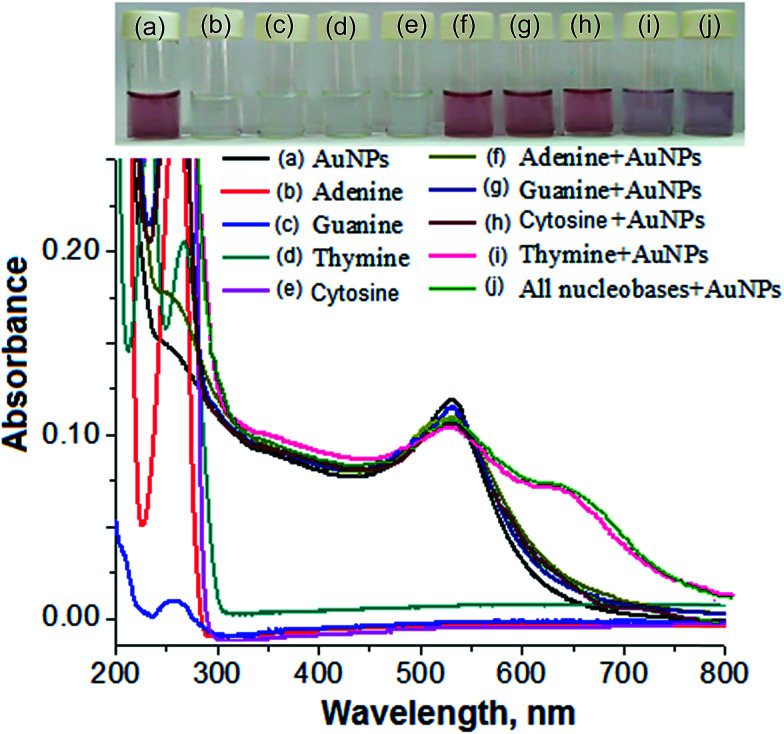
UV-Vis spectra of (a) the AuNPs, and the AuNPs with a fixed concentration (500 ng mL^−1^) of nucleobase (b) adenine, (c) guanine, (d) thymine and (e) cytosine in the absence of the AuNPs, as well as of (f) adenine + AuNPs, (g) guanine + AuNPs, (h) cytosine + AuNPs, (i) thymine + AuNPs and (j) all nucleobases + AuNPs.

Therefore, we performed separate sets of control experiments with individual nucleobases and mixtures of nucleobases in the presence and absence of the AuNPs. All of the individual nucleobases showed an absorption peak in the UV region in the absence of the AuNPs, as shown in Fig. 4(b)–(e). No color change or red shift of the LSPR absorption peak were obtained for adenine, guanine and cytosine in the presence of the AuNPs, revealing that there was no interaction or replacement of the surface-stabilized molecules by these nucleobases, as shown in Fig. 4(f)–(h). The color change and red shift of the AuNPs were exhibited in the presence of thymine because the structural orientation of the molecule was found to be suitable for interaction with gold, followed by the replacement of DBS from the surface of the AuNPs, as shown in Fig. 4(i). Furthermore, a similar color change from pink to blue and a similar red shift of the LSPR absorption peak as were obtained for thymine in the presence of the AuNPs were acquired after the addition of all four nucleobases. This concluded that the AuNPs could act as a selective biochemical sensor for the detection of thymine in the presence of other nucleobases in a sample solution (Fig. 4(j)).

**Fig. 5:**
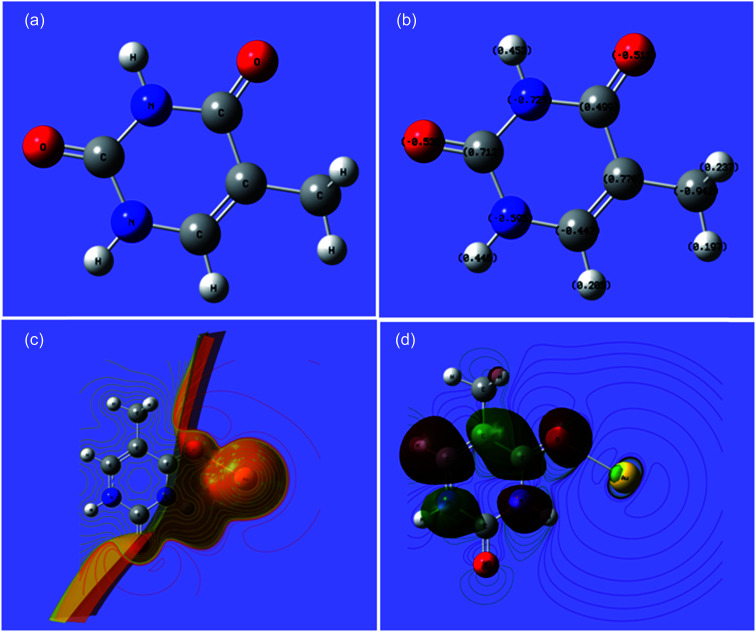
(a) The optimized labeled structure of thymine, (b) thymine showing charge distribution at each atom, (c) the interaction of thymine with Au-ESP and (d) the HOMO–LUMO of thymine and gold.

Furthermore, a theoretical study to show the interaction of thymine with gold and all of the individual biomolecules was performed using density functional theory (DFT) calculations using the Gaussian 09 (C.01) program with the B3LYP method and LANL2DZ basis set.^36*a*^ The results obtained (Cartesian coordinates, bond parameters and energy values, and charge distributions) are shown in the ESI (Tables S2–S8, Fig S3 and S4).† The interaction of gold with thymine is shown in Fig. 5(a)–(c). The bond length of Au–O, bond angle of Au–O–C and dihedral angle of Au–O–C–N were 2.4999 Å, 122.5588° and 0.0273°, respectively. The total energy was calculated to be −589.5187 atomic units (Table S1†). The optimized structure and charge distribution on each atom in thymine are shown in Fig. 5(a) and (b). The electrostatic potential (ESP) provided very important information about the reactive behavior of the molecule, especially in non-covalent interactions. ESP is the force acting on a positive test charge (a proton) located at a particular point in the electrical charge cloud generated by the molecule’s electrons and nuclei, as shown in Fig. 5(c). The energy gap between the HOMO and LUMO was found to be 3.17 eV (Fig. 5(c)), which is also known as a frontier orbital. Furthermore, the omnicapped truncated tetrahedral (*ν*_3_-tetrahedral) Au_20_ cluster structure was considered as the model for the AuNP optimization and its interaction with thymine,^36*b*^ and is shown in the ESI (Fig. S5 and Table S9).† It depicts the stronger non-covalent interaction between a AuNP and thymine (Fig. 5 c). All these data were found to be consistent with the interaction or non-covalent bond formation of gold with oxygen, as well as with the FTIR analysis of a pure thymine molecule and a solution mixture of the AuNPs with thymine, as shown in Fig. 2(d). A decrease in signal intensity and a shift of the IR band from 1661 to 1610 cm^−1^ for C=O and 1050 to 1040 cm^−1^ for C–O confirmed the binding of an oxygen atom from the thymine molecule to the surface of a AuNP through non-covalent interactions. The similar shift of the IR bands for a free thymine and bound thymine molecule on the surface of a AuNP has been reported by Avvakumova *et al.*, and this verifies the binding or interaction of the analyte on the surface of the AuNPs.^32^ Thus, the results obtained from the theoretical investigation demonstrated the possibility of the interaction of gold with other atoms of thymine might rule out structural orientation, energy and affinity as the reason for the detection. Furthermore, the AuNPs can be used as a selective biochemical sensor for the determination of thymine from biological samples.

### Analytical evaluation for the determination of thymine using the AuNPs as a biochemical sensor

**Fig. 6:**
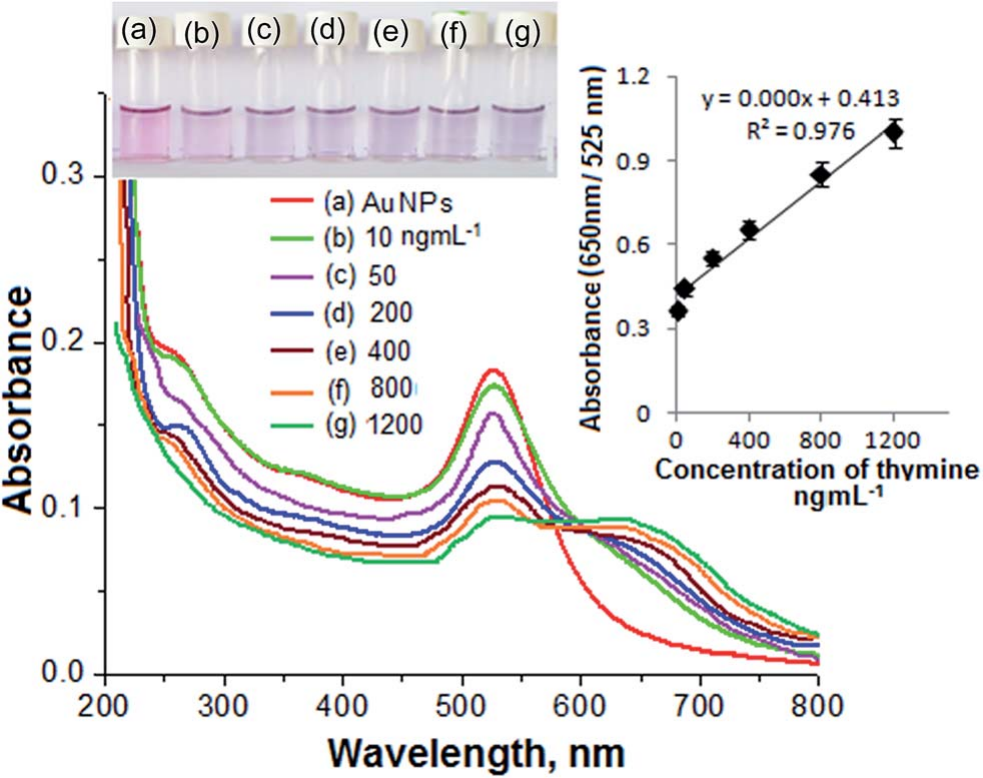
Images of glass vials containing the DBS–AuNPs and different concentrations of thymine (10, 50, 100, 200, 400, 800 and 1200 ng mL^−1^) using the DBS–AuNPs as a colorimetric probe at pH 7.0 for 10 min of reaction time at room temperature, along with the respective UV-Vis spectra.

The linearity range, correlation coefficient, limit of detection (LOD) and precision for the determination of thymine were estimated to determine the plausibility of using the AuNPs as a biochemical sensor. The calibration curve for thymine was determined by spiking different concentrations of thymine (10, 50, 100, 400, 800 and 1200 ng mL^−1^) into separate glass vials containing 1.0 mL of the AuNPs and the total volume was maintained at 1.0 mL with DW. The results are given in Fig. 6. The color change and signal intensity of the solution mixture were dependent upon the degree of aggregation of the NPs and were found to be directly proportional to the concentration of the analyte added into the solution mixture. Good linearity was observed over the range 10–1200 ng mL^−1^ for the determination of thymine with a correlation coefficient (*R*^2^) of 0.997. The LOD was estimated by spiking a minimum quantity of the analyte into the NP solution and using three times the standard deviation with the slope of the curve (3*σ*/slope). The LOD obtained for the determination of thymine was 3.5 ng mL^−1^.

The precision for the determination of thymine using the AuNPs as a biochemical sensor was estimated by calculating the relative standard deviation percentage (RSD, %) by analysing six replicate samples of an analyte (100 ng mL^−1^) under the optimized conditions. The RSD acquired for the determination of thymine was 2.5%, revealing a better stability of the AuNPs for use as a biochemical sensor.

### Effect of chemical interferences for the detection of thymine in urine samples using the AuNPs as a biochemical sensor

**Fig. 7:**
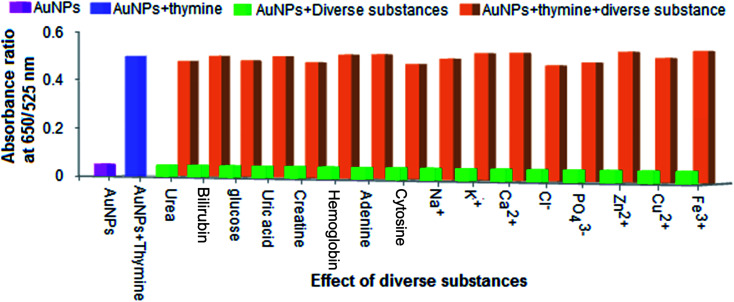
Effect of the absorbance ratio (at 650/525 nm) of biomolecules, metal ions and anions in the presence and absence of the AuNPs for selective determination of thymine. The concentrations of the diverse substances such as bilirubin, glucose, uric acid, and creatine (600 mg L^−1^), haemoglobin, guanine, adenine, and cytosine (450 mg L^−1^), Na^+^, K^+^, Fe^3+^, Ca^2+^, Cl^−^, and PO_4_^3−^ (850 mg L^−1^) and Zn^2+^ and Cu^2+^ (700 mg L^−1^) were spiked into separate glass vials containing the AuNPs, and the pH of the sample solution (pH 7.0) and the reaction time (5 min) were maintained.

The chemical substances, such as urea, bilirubin, glucose, uric acid, creatine, haemoglobin, adenine, cytosine, Na^+^, K^+^, Ca^2+^, Zn^2+^, Cu^2+^, Fe^3+^, Cl^−^ and PO_4_^3−^, that might exist in urine samples were investigated with regard to the selective detection of thymine using the AuNPs as a biochemical sensor. For this, different separate sets of experiments were carried out by spiking the diverse substances of urea, bilirubin, glucose, uric acid, and creatine (600 mg L^−1^), guanine, adenine, and cytosine (450 mg L^−1^), Na^+^, K^+^, Fe^3+^, Ca^2+^, Cl^−^, and PO_4_^3−^ (850 mg L^−1^) and Zn^2+^and Cu^2+^ (700 mg L^−1^) into separate glass vials containing the AuNPs and maintaining the pH of the sample solution (pH 7.0) and the reaction time (5 min) within their respective tolerance limits. The result is shown in Fig. 7. The ratio of absorbance (at 650 nm to 525 nm) is shown on the *y*-axis and the effect of the diverse substances is given on the *x*-axis. The light green bars in the diagram represent the signal response of the AuNPs + diverse substances and the pink bar shows the signal response of the AuNPs alone. Next, an experiment was performed by adding thymine and different diverse substances (within their respective tolerance limits) into the AuNP solution, and the results are shown as light brown bars in the diagram. Consequently, the signal response of the AuNPs with thymine (the light blue bar) shows the same response as the solution mixture of AuNPs + thymine + diverse substances. From these results, it can be seen that the absorbance ratio of thymine exhibited the same response as was observed for the diverse substances and thymine in the presence of the AuNPs. This revealed that the presence of the diverse substances did not affect the determination of thymine using the AuNPs as a biochemical sensor at a particular tolerance limit.

### Application for the determination of thymine in urine samples using an LSPR-based biochemical sensor

The developed method was applied for the qualitative and quantitative determination of thymine in a DNA standard and urine samples using the AuNPs as a biochemical sensor, and the results obtained were compared with those from LC-MS analysis.^37^ The qualitative determination of thymine was demonstrated by spiking the diluted hydrolyzed product of the DNA standard into the AuNP solution, as shown in Fig. S6.† The intensity of the color change of the solution from pink to blue increased with an increasing concentration of the hydrolyzed product (thymine), and the result could be seen by the naked eye. The quantitative determination of thymine in urine samples was performed by mixing an aliquot of the urine sample and the AuNPs while other experimental conditions were kept constant. The results are given in Table 1. The thymine present in the urine samples was found to be in the range 46.6–101.2 ng mL^−1^. The accuracy of the method was calculated by spiking thymine (100 and 200 ng mL^−1^) into a glass vial containing the urine sample, and the experiments were performed with pH 7.0 and a reaction time of 5 min. A recovery percentage from 93.5 to 96.3% (Table 2) was obtained in the urine sample, showing the good accuracy of the results for the determination of thymine using the AuNPs as a biochemical sensor.

**Table 1:** Application of the determination of thymine in urine samples using the AuNPs as a biochemical sensor

Samples	AuNPs/colorimetry		LC-MS	
	Thymine, ng mL^−1^	RSD, %	Thymine, ng mL^−1^	RSD, %
Urine-1	46.6	2.1	45.3	3.5
Urine-2	101.2	1.8	115.3	2.8
Urine-3	67.5	2.4	72.5	3.1

**Table 2:** Recovery percentage (%) for the determination of thymine in urine sample-1 using the AuNPs as a biochemical sensor

Found using present method, ng mL^−1^	Added	Recovered	Recovery, %	RSD, %
48.5	100	143.1	96.3	2.5
	200	232.5	93.5	3.1

### Comparison of the AuNP-based colorimetric method for the determination of thymine with other reported methods

The potential for using the AuNPs as a biochemical sensor for the determination of thymine was evaluated by comparing the linearity range and LOD of GC,^4^ LC-MS,^37^ glass carbon electrode-linear sweep voltammetry (GCE-LSV),^7^ silicon carbon nanoparticles modified glassy carbon electrode-differential pulse voltammetry (SiCNPs/GCE-DPV),^38^ and titanium dioxide nanoparticles-magnesium doped zeolite Y modified carbon paste electrode-differential pulse voltammetry (TiO_2_NPs-MgY/ZMCPE-DPV)^39^ (Table 3). The present method showed a high sensitivity for the detection of thymine compared to GC, GCE-LSV and SiCNP/GCE-DPV. The use of TiO_2_NPs-MgY/ZMCPE with DPV exhibited a better LOD for the determination of thymine, but the synthesis of the TiO_2_ NPs followed by the modification of an electrode with the NPs was found to be a tedious and complex process. The running costs of GC and LC-MS methods are expensive because they require high purity reagents and organic solvents for a long amount of time for chromatographic separation. In addition, electrochemical methods require sophisticated, expensive electrodes, and the surface modification of an electrode is a complex and time consuming process for the redox detection of analytes from complex sample matrices. Therefore, the present method is simple, rapid, sensitive and cost effective compared to chromatographic and electrochemical methods for the determination of thymine from urine samples.

**Table 3:** Determination of thymine using the AuNPs as a biochemical sensor compared to other reported methods

Analytical methods	Linear range, μM	LOD, μM	Ref.
GC, GCE-LSV, SiCNP/GCE-DPV	0.5–50	0.09	4
LC-MS	0.016–3.95	0.026	37
GCE-LSV	100–2300	110	7
SiCNP/GCE-DPV	1.2–136	0.14	38
TiO_2_NPs-MgY/ZMCPE-DPV	0.1–10	0.013	39
AuNPs/colorimetry	0.079–7.21	0.027	Present method

### Future perspective for the application of the AuNPs as a biochemical sensor for the determination of uracil

Furthermore, we also tested the determination of uracil using the AuNPs as a biochemical sensor under the optimized conditions by spiking different concentrations of uracil (50, 100, 250, 500 and 750 ng mL^−1^) into a glass vial containing 1 mL of the AuNPs, and the total volume of the solution mixture was made up to 2 mL with DW. The results are shown in the ESI in Fig. S7.† The color of the solution mixture changed from pink to blue and there was a red shift of the LSPR band in the visible region, as with the introduction of thymine into the NP solution. This is due to the similar chemical structure of uracil to that of a thymine molecule. However, the peak at 278 nm in the UV range as well as the red shift from 525 nm to 580 nm for uracil could be specifically used for the selective determination of uracil from a sample solution. The linearity range was found to be 50–750 ng mL^−1^ with an *R*^2^ value of 0.984 for the determination of uracil. Hence, in the near future, the AuNPs could be also used as a biochemical sensor for the determination of uracil in biological samples.

## Conclusions

DBS-capped AuNPs were successfully demonstrated for use as a label-free biochemical colorimetric sensor for the qualitative and quantitative determination of thymine in a DNA standard and in urine samples in the presence of other nucleobases. The sensing mechanism for the selective detection of thymine was theoretically and experimentally elucidated to verify that the interaction between thymine and gold is due to the electrostatic potential behavior and spatial arrangement of the atoms in the molecule. The employment of the AuNPs as a biochemical sensor is label-free, simple, and economic and the sensor can be applied at the sample source for the determination of thymine in biological samples. In the near future, this colorimetry-based biochemical sensor will be highly useful for the detection of thymine in samples like blood and serum for clinical diagnosis.

## Conflicts of interest

There are no conflicts to declare.

## Supplementary Material

RA-008-C8RA02627K-s001
