# Supplementary material for: Experimental and theoretical approaches for the selective detection of thymine in real samples using gold nanoparticles as a biochemical sensor
Source: RSC Adv. 2018 Jul 5;8(43):24328–37. doi: 10.1039/c8ra02627k (PMC9082146; doi:10.1039/c8ra02627k)
Supplement: RA-008-C8RA02627K-s001 [file RA-008-C8RA02627K-s001.pdf]

Electronic Supplementary Material (ESI) for RSC Advances.
This journal is © The Royal Society of Chemistry 2018

Electronic Supplementary Information:

Experimental and theoretical approach for selective detection of thymine in real samples using gold nanoparticles as a biochemical sensor

Kamlesh Shrivas[1,2*] Nidhi Nirmalkar[2], Santosh Singh Thakur[2], Ramsingh Kurrey[3], Deepak Sinha[4] and Ravi Shankar[5]

[1]School of Studies in Chemistry, Pt. Ravishankar Shukla University, Raipur, CG-492010, India

[2]Department of Chemistry, Guru Ghasidas Vishwavidyalaya, Koni, Bilaspur, CG

[3]School of Studies in Chemistry, Pt. Ravishankar Shukla University, Raipur-492010, Chhattisgarh, India

[4]Department of Chemistry, Government Nagarjuna Post Graduate College of Science, Raipur, CG-492010, India

[5]Nanoscience and Nanoengineering Program, South Dakota School of Mines and Technology, Rapid City, South Dakota-57701, USA

***Corresponding author: Email:kshrivas@gmail.com**

Phone: +91-7752-260488

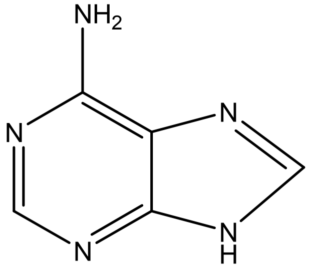

Adenine

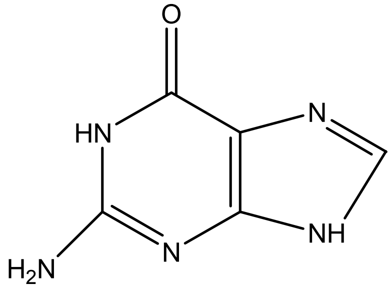

Guanine

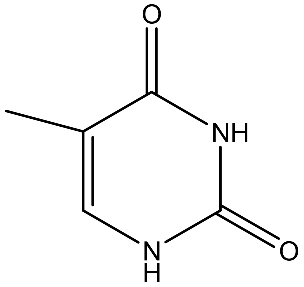

Thymine

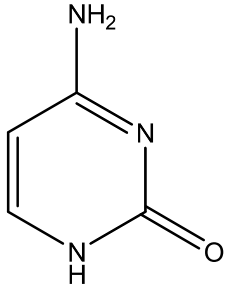

Cytosine

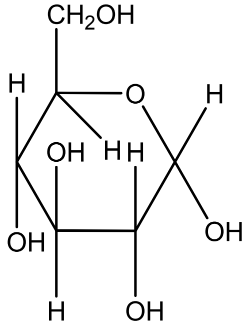

Glucose

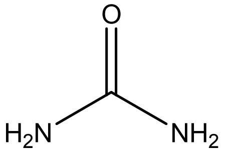

Urea

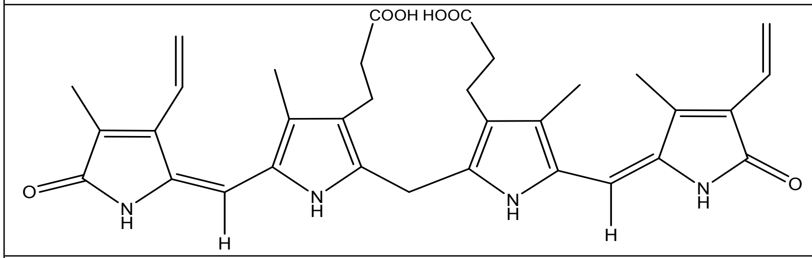

Bilirubin

Fig. S1. Structure of chemical substances

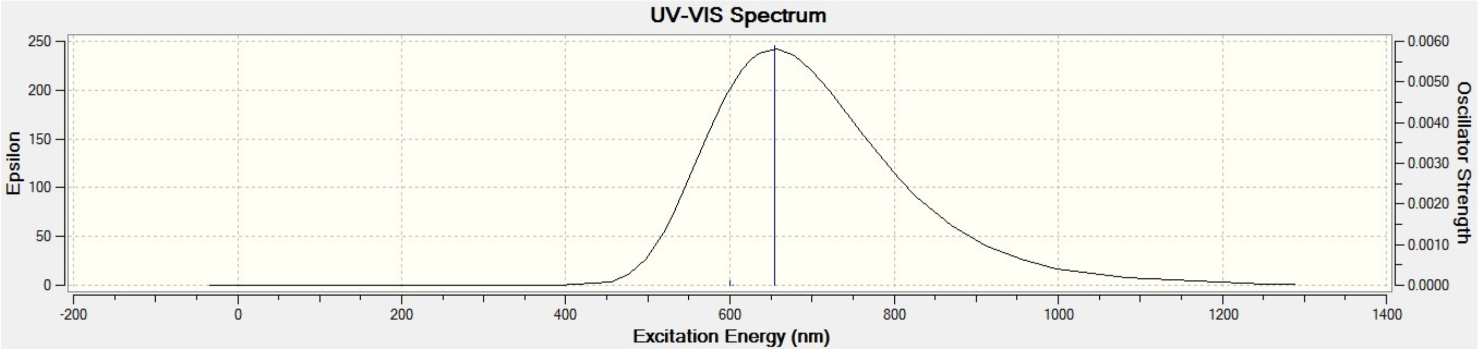

Fig. S2 TD-DFT of AuNPs with thymine calculated using Polarizable Continuum Model (PCM) for the solvent water and integral equation formalism variant (IEFPCM) in self consistent filed method (B3LYP method & LANL2DZ basis set).

Table S1. Absorption band of AuNPs with thymine by TD-DFT calculation

Solvent	Model	Absorption band in nm
Water	PCM	654.8
	CPCM	658.4
	iCEM	661.04
	iPCM	661.06
	SMD	666.0

Table S2. The Cartesian coordinates and bond parameters of thyamine gold interaction calculated by density functional theory (DFT) using B3LYP method and LANL2DZ basis set (E= -589.51873015 a.u.)

Row	Symbol	Bond	Angle	Dihedral	X	Y	Z
1	O				-0.2302	1.176443	0.0005781
2	O	4.654858			-2.55221	-2.85791	-0.0001415
3	N	2.319572	0.707818		-1.41203	-0.81948	0.0002931
4	N	2.328603	60.80532	0.148403	-3.74743	-0.85945	-0.0000096
5	C	2.408127	62.00087	179.8409	-2.63579	1.287011	-0.0000008
6	C	1.26869	31.35862	179.8443	-1.35028	0.580621	0.0003476
7	C	1.367187	143.7642	0.007657	-3.77449	0.530339	-0.000118
8	C	1.507528	92.28868	-179.993	-2.62676	2.794512	-0.0002687
9	C	1.246759	30.59026	-0.00616	-2.56673	-1.61123	0.000032
10	H	1.084678	121.8056	179.9927	-4.7608	0.981702	-0.0003159
11	H	1.022444	88.51574	179.9821	-0.51905	-1.31745	0.0001284
12	H	1.013883	88.65639	179.9986	-4.60512	-1.40011	-0.0001825
13	H	1.096747	110.7858	59.07446	-2.09728	3.180402	-0.8798087
14	H	1.096736	110.7874	-59.035	-2.0971	3.180737	0.8789981
15	H	1.095781	111.1987	-179.981	-3.64601	3.196859	-0.0002401
16	Au	2.499972	122.5588	0.027298	1.947153	-0.05197	-0.0000508

Table S3. The Cartesian coordinates and bond parameters of adenine calculated by density functional theory (DFT) using B3LYP method and 6-31+G(d) basis set (E= -467.25962349 a.u.)

Row	Symbol	Bond	Angle	Dihedral	X	Y	Z
1	N				2.034903	-0.361843	0.035194
2	C	1.4946354			1.489325	-1.753185	0.014049
3	N	1.3109426	121.5565605		0.199221	-1.983825	-0.017688
4	C	1.4621031	118.2172519	-0.1194	-0.707943	-0.837239	-0.029505
5	C	1.3127704	121.2836677	4.72112	-0.252281	0.39181	-0.101456
6	C	1.3016895	120.9697972	-2.5903	1.242883	0.669243	-0.027746
7	N	1.4700004	121.4487981	-178.82	1.769523	2.041656	-0.033591
8	N	1.4681389	128.8772251	-173.93	-2.173409	-0.820744	0.057496
9	N	1.4600779	109.834637	173.719	-1.365637	1.325276	-0.246049
10	C	1.3113039	105.3678617	175.054	-2.503966	0.446977	0.113473
11	H	1.0700002	119.2218531	177.534	2.167713	-2.580542	0.027105
12	H	0.9999992	109.4712018	-150.14	1.103514	2.656062	0.389417
13	H	0.9999996	109.4711683	-30.142	2.625789	2.072309	0.482033
14	H	1.0000004	113.6566051	136.316	-1.283787	2.13954	0.328647
15	H	1.0699995	124.1629312	-166.22	-3.473557	0.803355	0.392376

Table S4. The Cartesian coordinates and bond parameters of cytosine calculated by density functional theory (DFT) using B3LYP method and 6-31+G(d) basis set (E= - 394.90515340 a.u.)

Row	Symbol	Bond	Angle	Dihedral	X	Y	Z
1	C				0.9090589	1.273304	-0.103144
2	C	1.3549062			-0.3644112	1.7348837	-0.071697
3	N	1.4754641	121.1234414		-1.4852597	0.8550808	0.3112387
4	C	1.474523	111.7667978	-25.6054526	-1.1819081	-0.5634683	0.0468257
5	N	1.4703638	121.1050844	27.6451234	0.202521	-1.0586883	0.0559389
6	C	1.2886386	120.2332021	-13.6297026	1.1884938	-0.2292437	0.0342916
7	N	1.4700001	120.2439992	176.4022839	2.5712162	-0.7202698	0.1230192
8	O	1.2584001	119.4426058	-152.8093413	-2.1243565	-1.3653125	-0.182102
9	H	1.0700001	119.9168927	-170.2657495	1.7217696	1.9576758	-0.22978
10	H	1.0700001	119.4445721	-170.4496428	-0.5596934	2.7570487	-0.320582
11	H	1.0000001	108.9448146	-146.2882053	-2.29755	1.119091	-0.208841
12	H	1.0000001	109.4712208	29.6018724	2.6262138	-1.6354544	-0.276246
13	H	1.0000001	109.4712211	149.6018722	3.1773692	-0.1015741	-0.376775

Table S5. The Cartesian coordinates and bond parameters of glucose calculated by density functional theory (DFT) using B3LYP method and 6-31+G(d) basis set (E= -687.16520376 a.u.)

Row	Symbol	Bond	Angle	Dihedral	X	Y	Z
1	C				1.1862307	-1.4220962	-0.2097575
2	C	1.5403513			1.6949879	-0.0048566	-0.5342262
3	C	1.5307537	108.6551133		0.8172782	1.0108274	0.2014494
4	C	1.53076	108.0443539	-58.5559624	-0.6289619	0.8079418	-0.2573024
5	C	1.540368	108.6556407	58.5630905	-1.0467426	-0.6411045	0.0564776
6	O	1.4394424	109.8976168	59.8172041	-0.181924	-1.5607941	-0.6350926
7	O	1.4300001	109.8915495	179.9219418	3.0535965	0.1333691	-0.1099898
8	O	1.4300001	110.096523	-178.821989	1.2465463	2.3410308	-0.1005244
9	O	1.4300001	109.7573849	178.7495729	-1.4871062	1.7215804	0.430995
10	C	1.5400001	109.2443197	-179.790067	-2.4993724	-0.8617466	-0.4048077
11	O	1.4300001	109.4712206	-59.8614038	-3.361472	0.0482132	0.2834203
12	O	1.4300001	109.3451374	58.3608711	1.2690734	-1.6465694	1.2000826
13	H	0.9600001	109.4712208	60.2701427	3.5994288	-0.50835	-0.5702765
14	H	0.9600001	109.4712209	-60.4907417	2.1562613	2.4559956	0.1837324
15	H	0.9600001	109.4712204	-180	-4.2670075	-0.08933	-0.0041342
16	H	0.9600001	109.4712207	-179.703272	-2.3932853	1.587705	0.1437374
17	H	0.9600001	109.4712207	179.7938387	2.1814347	-1.5576677	1.4852027
18	H	1.0700001	109.5328882	-119.812996	1.7914555	-2.1402002	-0.7225244
19	H	1.0700001	109.5208602	179.9672018	1.6340324	0.1641313	-1.5890379
20	H	1.0700001	109.6657325	60.9340895	0.8863536	0.8487739	1.2568486
21	H	1.0700001	109.6528356	-61.1121667	-0.6998144	0.9803002	-1.3109497
22	H	1.0700001	109.455967	60.2796646	-0.9761196	-0.8121149	1.11036
23	H	1.0700001	109.4712209	60.1385963	-2.5687587	-0.6908303	-1.4587874
24	H	1.0700001	109.4712204	-179.861403	-2.7942131	-1.8668452	-0.1862991

Table S6. The Cartesian coordinates and bond parameters of guanine calculated by density functional theory (DFT) using B3LYP method and 6-31+G(d) basis set (E= -542.49883324 a.u.)

Row	Symbol	Bond	Angle	Dihedral	X	Y	Z
1	N				1.6250048	0.9479212	-0.3121434
2	C	1.4972051			1.7959029	-0.5136213	-0.0358995
3	N	1.3145754	123.2243588		0.8039772	-1.375292	-0.07732
4	C	1.4622908	119.8911515	-14.9739119	-0.5777038	-0.8964899	-0.0779564
5	C	1.3096361	121.6417609	3.0186373	-0.8618443	0.3818128	-0.0967453
6	C	1.4786286	112.514342	25.2306229	0.2478693	1.4168759	-0.047716
	N	1.4700001	118.3841676	-155.0656082	3.1401024	-1.0104148	0.2915515
8	O	1.2584001	121.3282922	157.0958538	0.0039601	2.6276232	0.1934684
9	N	1.460506	110.0309657	177.2851004	-2.3086595	0.571849	-0.1574911
10	N	1.4682435	128.4611174	-174.4079645	-1.8163451	-1.6817095	-0.0076949
11	C	1.3110863	105.3189134	171.8116634	-2.7765925	-0.8013974	0.1403684
12	H	1.0000001	108.9027671	36.0489683	2.2593249	1.4653179	0.2622595
13	H	1.0000001	109.471221	-149.8591561	3.2312931	-1.9559565	-0.0209152
14	H	1.0000001	109.4712201	-29.8591568	3.8255761	-0.4415835	-0.1629337
15	H	1.0000001	113.6799471	134.6550099	-2.6495515	1.2658829	0.4766301
16	H	1.0700001	124.1777878	-165.3547537	-3.7726713	-1.0442049	0.44659

Table S7. The Cartesian coordinates and bond parameters of urea calculated by density functional theory (DFT) using B3LYP method and 6-31+G(d) basis set (E= -225.25786004 a.u.)

Row	Symbol	Bond	Angle	Dihedral	X	Y	Z
1	C				0	0	0.1300875
2	N	1.47			0	1.2730575	-0.604913
3	N	1.47	120		0	-1.2730575	-0.604913
4	O	1.258	120	180	0	0	1.3884876
5	H	1	120	0	0	2.1390829	-0.104913
6	H	1	120	180	0	1.2730575	-1.604913
7	H	1	120	0	0	-2.1390829	-0.104913
8	H	1	120	-180	0	-1.2730575	-1.604913

Table S8. The Cartesian coordinates and bond parameters of bilirubin calculated by density functional theory (DFT) using B3LYP method and 6-31+G (d) basis set (E= -1949.58777280 a. u.)

Row	Symbol	Bond	Angle	Dihedral	X	Y	Z
1	O				-6.9809395	-0.8249639	0.6663834
2	O	4.1051209			-3.8796038	-0.411106	3.3239117
3	O	2.2692189	133.0000882		-7.0851066	-0.8395122	-1.6003967
4	O	2.2692892	62.9472978	-91.7186173	-4.4315261	-2.5227345	2.7025579
5	O	6.0308647	126.9347053	40.7997018	0.7841752	3.387607	2.8877089
6	O	5.1615942	106.6665029	-98.8447625	4.742632	0.6617888	1.0055321
7	N	5.7112169	62.3307218	-44.3456077	-0.746813	0.517688	-1.8067173
8	N	3.3141314	103.7631056	-79.7144987	0.6022729	-2.4783832	-1.3742919
9	N	2.3454981	72.2747546	-29.013468	0.0838475	2.8638083	0.7113502
10	N	2.3450806	94.3339122	14.3180905	3.4080599	-1.1429952	0.3265068
11	C	2.4782027	48.0369655	128.7321136	-1.702208	-1.7649822	-1.9416679
12	C	1.3469148	122.7368114	6.8412079	-1.8506302	-0.2521726	-1.86223
13	C	1.370481	63.1508407	96.0261765	-0.7102891	-2.3319097	-1.0083071
14	C	1.3471437	111.0412638	69.9904016	-2.9673451	0.5008707	-1.8363503
15	C	1.377446	107.8378884	-127.9928369	-0.8416896	-2.7903461	0.2839496
16	C	1.4168083	105.8788761	0.0134775	-2.5266683	1.8451782	-1.7589748
17	C	1.416905	107.3729316	0.0173899	0.4370449	-3.2274269	0.7098817
18	C	1.3705779	63.9300611	102.0913002	-1.1495639	1.8261814	-1.7425251
19	C	1.3569695	106.482811	0.0049415	1.2819926	-3.0179079	-0.3310464
20	C	1.4775244	127.6743369	179.937367	-4.3698177	0.0380596	-1.8805637
21	C	1.4774076	126.171992	179.9372339	-2.087917	-2.8177256	1.0769824
22	C	1.4778964	126.2943215	-179.9797307	-3.3865545	3.0459751	-1.7054918
23	C	1.4778174	126.2855591	-179.9772682	0.7708343	-3.7980957	2.0315723
24	C	1.542028	114.4856824	90.0852867	-4.9961166	-0.2364652	-0.4984507
25	C	1.5421043	114.4886546	-89.9155263	-2.3477996	-1.542017	1.9034896
26	C	1.4041904	117.9425584	61.7408592	-0.1572855	2.8176724	-1.6786512
27	C	1.4334998	133.3228027	179.9217964	2.6840652	-3.2478734	-0.521388
28	C	1.3545323	125.4926707	-88.3369082	0.4081761	3.2923906	-0.5430222
29	C	1.3357854	124.6228933	-88.2880141	3.6453875	-2.3732903	-0.2127032
30	C	1.4486418	129.5418661	-179.9982383	1.4301883	4.305265	-0.3752733
31	C	1.4547455	129.3941589	179.9962524	5.0874219	-2.4962077	-0.3600557
32	C	1.222897	30.1777756	0.9727534	-6.4443798	-0.6702623	-0.5726318
33	C	1.2229654	30.1803068	109.7997443	-3.6502624	-1.5822915	2.6735986
34	C	1.3506336	109.1317772	179.9877577	5.6757314	-1.3638725	0.0825727
35	C	1.3470446	110.0847371	-179.9983404	1.7045798	4.4733707	0.9327706
36	C	1.2186439	99.6361889	-25.9685441	0.82215	3.5258777	1.6775304
37	C	1.2184335	121.5769446	11.7762025	4.5857143	-0.4538536	0.5415289

38	C	1.4924044	121.8098535	0.0003938	2.0372778	5.010976	-1.5417574
39	C	1.4404283	126.9348825	-0.0411658	5.8105477	-3.6250196	-0.8870341
40	C	1.4852349	132.4637917	179.9801472	7.0965447	-0.9400139	0.1695001
41	C	1.4276948	130.2436202	-179.995564	2.6298428	5.3500981	1.575849
42	C	1.3332523	123.4149475	140.03132	6.8716159	-3.5092229	-1.6859628
43	C	1.3403412	123.8661581	40.0116173	2.8476786	6.6163797	1.1942821
44	H	1.0960952	131.7175799	-153.0904684	-2.6755127	-2.2391419	-1.7705678
45	H	1.0985438	85.4109154	100.3022706	-1.4315605	-2.0184481	-2.9757392
46	H	1.0118228	85.0344201	-124.3032843	0.2138203	0.2001832	-1.8193522
47	H	1.0118218	79.0777057	-124.8784022	0.9893439	-2.2217668	-2.2732399
48	H	1.0980365	109.4755245	-147.5978492	-4.9845761	0.7822428	-2.4039636
49	H	1.0978664	110.0056418	-33.1119411	-4.4470644	-0.8682034	-2.495406
50	H	1.0971649	109.938529	33.2904398	-2.9464408	-2.9999533	0.4185748
51	H	1.0980518	109.6027784	147.7291349	-2.07294	-3.6796851	1.7570702
52	H	1.0946348	112.1170931	173.1126274	-2.8091397	3.9587758	-1.527693
53	H	1.094922	110.4064905	-66.7504886	-3.9263603	3.1713569	-2.6498133
54	H	1.0949008	110.6940359	53.1593276	-4.121305	2.9600693	-0.8982932
55	H	1.0946373	112.1259649	-174.8961185	1.8451557	-3.9747431	2.1449749
56	H	1.0949556	110.4489905	65.0003277	0.2593611	-4.7555556	2.1750767
57	H	1.0949155	110.6499085	-54.9133298	0.4625456	-3.1199181	2.8339894
58	H	1.0958077	109.8747733	56.9960169	-4.9374548	0.6643789	0.1227041
59	H	1.0924317	109.0545246	-62.3286177	-4.4420524	-1.0408926	-0.009251
60	H	1.0955717	109.7759238	57.2546055	-2.3687507	-0.6693127	1.2415034
61	H	1.0961727	109.5163458	-61.2595233	-1.535242	-1.4017529	2.6257574
62	H	1.0874951	115.8029672	92.2746708	0.1611964	3.2308057	-2.6328712
63	H	1.0872973	115.7445821	91.8232176	2.9507504	-4.2105901	-0.9506561
64	H	1.0135377	100.5227	-6.5029335	-0.6095769	2.150856	0.9065947
65	H	1.0134683	100.539925	4.7617658	2.4854727	-0.7820811	0.5402633
66	H	1.0956818	110.6060398	-47.4041284	2.327179	4.2940553	-2.3179666
67	H	1.0947956	110.5052905	72.5186134	1.3232704	5.7180998	-1.9762072
68	H	1.0926354	113.0874355	-165.8173832	2.9489413	5.5560855	-1.2856768
69	H	1.0860768	117.3326576	-38.39262	5.4448309	-4.6151257	-0.6310968
70	H	1.0941492	110.4945897	96.938032	7.3664128	-0.3251212	-0.6943521
71	H	1.0955361	110.4609552	-143.6873117	7.2637938	-0.344091	1.0734376
72	H	1.0932785	112.558351	-25.2099864	7.784033	-1.787765	0.2322366
73	H	1.0862109	117.0904949	-141.2525642	3.1662626	4.9589125	2.4355477
74	H	0.9809217	111.8629175	0.5691838	-7.9237845	-1.0924835	0.6253469
75	H	0.9809673	70.4585414	170.2503309	-4.7303695	-0.4250522	3.8120716
76	H	1.0856711	119.672557	-179.3918078	7.3653718	-4.4022841	-2.0565412
77	H	1.0837747	123.5589821	-1.4931815	7.2653273	-2.5581124	-2.0250024
78	H	1.085427	119.8037872	179.5169719	3.5619869	7.2279125	1.7364472
79	H	1.0840543	123.0185292	1.2788611	2.3188557	7.0848528	0.3720576

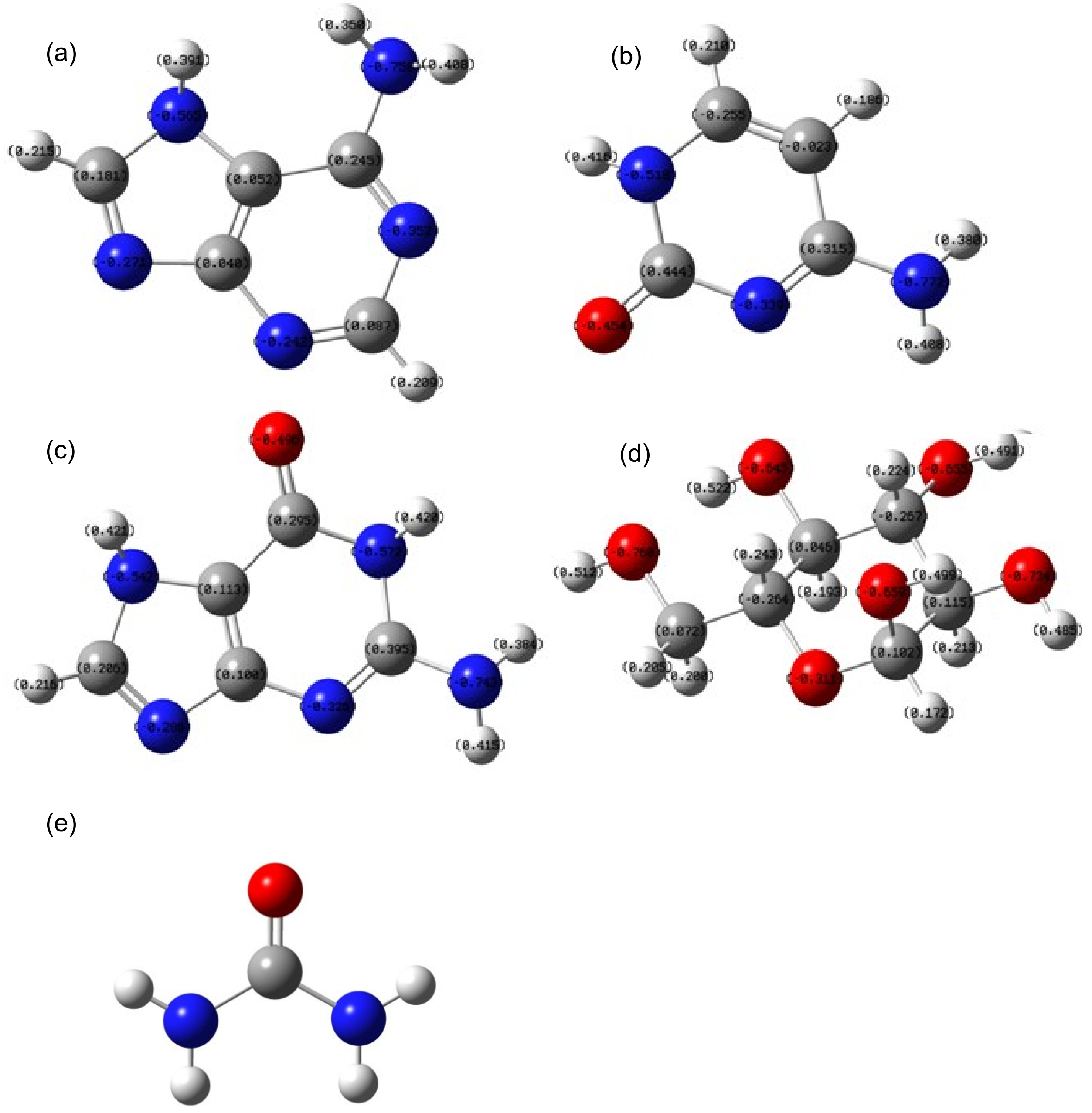

Fig. S3. Optimized structure of (a) adenine, (b) cytosine, (c) guanine, (d) glucose and (e) urea in the present study obtained from density functional theory (DFT) using B3LYP method and 6-31+G (d) basis set

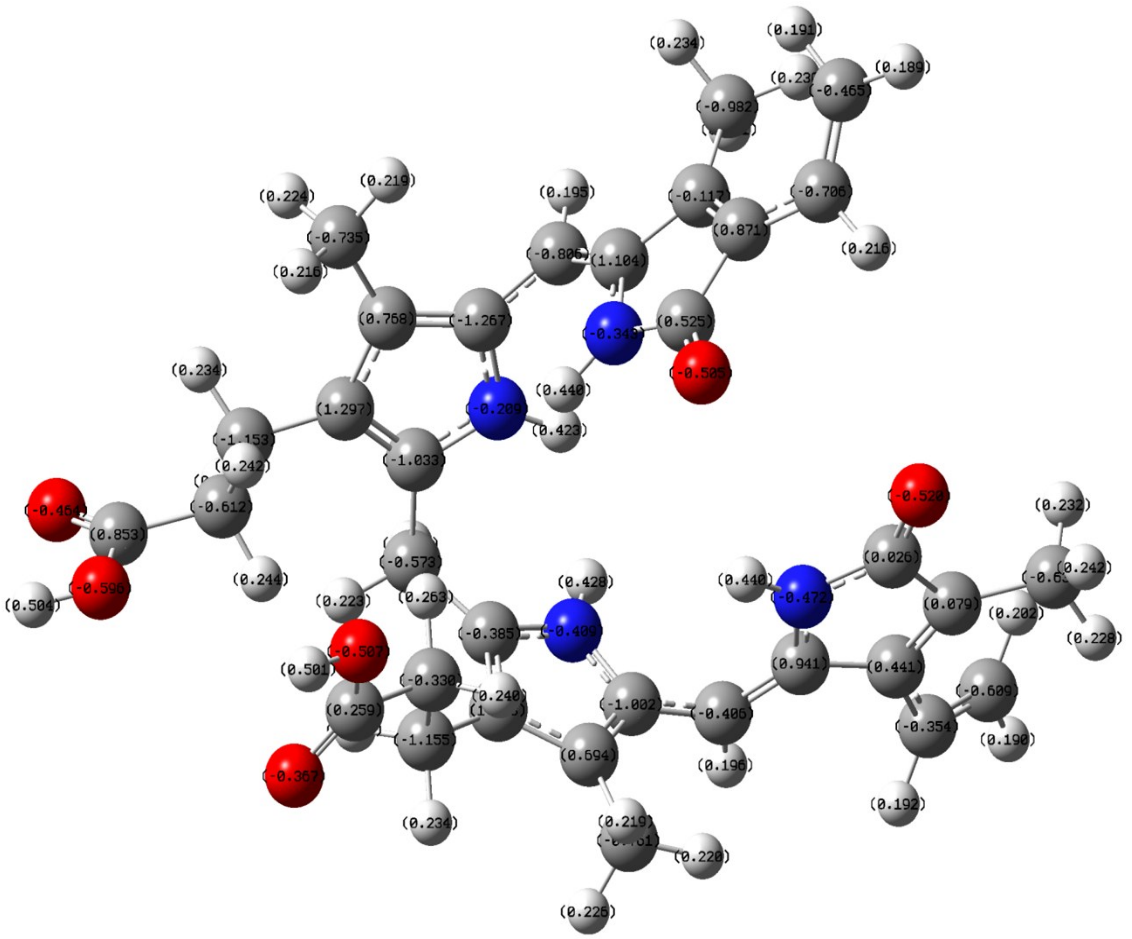

Fig. S4. Optimized structure of bilurubin taken in the present study obtained from density functional theory (DFT) using B3LYP method and 6-31+G (d) basis set.

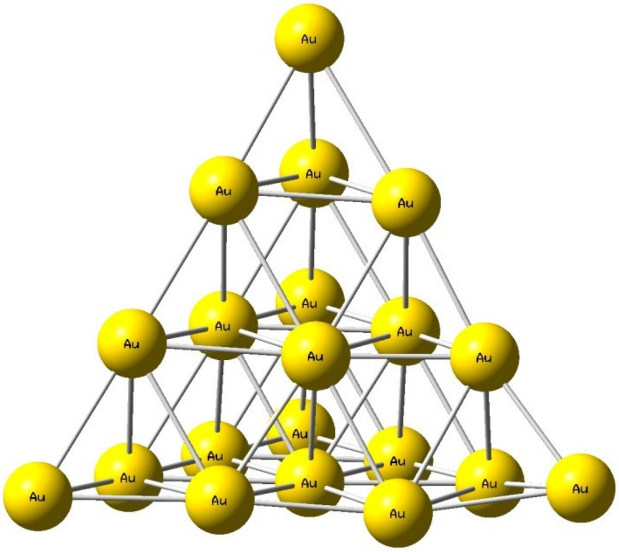

(a)

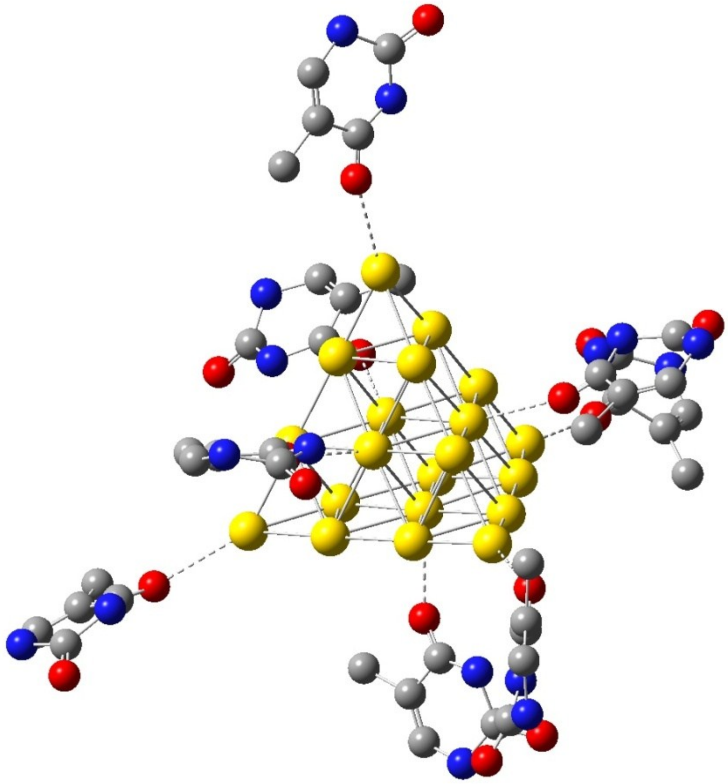

(b)

Fig. S5. (a) Optimized omnicapped truncated tetrahedral (ν_3-tetrahedral) Au_{20} cluster structure model (b) Omnicapped truncated tetrahedral (ν_3-tetrahedral) Au_{20} cluster interacted with thymine (hydrogen is not shown for clarity)-In both structure optimization is done using B3LYP method and LANL2DZ basis set.

Table S9. The Cartesian coordinates gold nanoparticles (Au_{20}) interacted with thymine calculated by density functional theory (DFT) using B3LYP method and LANL2DZ basis set (E= - -2709.74444741 a.u.)

Row	Symbol	X	Y	Z
1	O	4.810618	3.537719	-4.924586
2	O	8.316747	0.961335	-6.577398
3	N	6.222534	1.95086	-6.079545
4	N	8.290459	3.318847	-6.855543
5	C	6.655086	4.441377	-5.450655
6	C	5.824802	3.233678	-5.482918
7	C	7.841743	4.461535	-6.023578
8	C	6.006248	5.62131	-4.671069
9	C	7.640775	2.021604	-6.494772
10	H	8.467856	5.311499	-5.885601
11	H	6.081425	1.197684	-5.418016
12	H	9.270955	3.229633	-6.735026
13	H	6.097333	5.452023	-3.612665
14	H	4.961983	5.677737	-4.925785
15	H	6.484977	6.534722	-4.92723
16	Au	2.903248	2.018234	-3.330425
17	Au	1.466701	2.451638	-1.161162
18	Au	2.675081	0.101921	-1.508523
19	Au	0.585919	0.699241	-3.034324
20	Au	-0.872774	1.191972	-0.852271
21	Au	-1.832283	-0.574708	-2.619568
22	Au	1.263995	0.565809	0.734928
23	Au	0.013862	2.909393	1.029438
24	Au	0.386701	-1.228208	-1.135847
25	Au	2.507086	-1.772325	0.428674
26	Au	-3.242538	-0.128191	-0.402021
27	Au	-1.947485	-2.468848	-0.762071
28	Au	-4.147012	-1.927184	-2.127844
29	Au	-2.325328	1.71699	1.333639
30	Au	-1.124403	-0.730734	1.095474
31	Au	-1.436877	3.444266	3.181663
32	Au	-0.301936	1.047112	2.909311
33	Au	0.189286	-3.055201	0.847076
34	Au	1.049138	-1.281543	2.6206
35	Au	2.334243	-3.58776	2.35016
36	O	-1.595824	5.711704	4.988584
37	O	-4.943652	8.680295	5.793803
38	N	-3.749424	6.663989	5.470394
39	N	-3.249297	8.32037	7.420174

40	C	-1.426274	6.947332	6.613034
41	C	-2.332715	6.378346	5.606813
42	C	-1.820812	7.927923	7.38727
43	C	-0.000931	6.302987	6.632355
44	C	-4.022591	7.924197	6.198385
45	H	-1.100586	8.430192	7.99049
46	H	-3.990871	6.759696	4.494841
47	H	-3.279622	9.308502	7.513672
48	H	0.559156	6.627148	5.775105
49	H	-0.097079	5.229364	6.599635
50	H	0.513333	6.584271	7.520837
51	O	3.926821	-5.662018	3.58698
52	O	8.507361	-5.399546	4.00749
53	N	6.200398	-4.823123	4.074329
54	N	7.232478	-6.757552	5.483736
55	C	4.887824	-6.903604	4.883757
56	C	5.007455	-5.689349	4.123873
57	C	5.935528	-7.476416	5.419564
58	C	3.419903	-7.408345	4.930052
59	C	7.366762	-5.646128	4.488502
60	H	5.835539	-8.459918	5.807214
61	H	6.343502	-4.462499	3.14338
62	H	7.949075	-7.426451	5.317771
63	H	3.13705	-7.785816	3.966097
64	H	2.770924	-6.582203	5.18407
65	H	3.320524	-8.171852	5.659053
66	O	-9.756123	-2.748057	-5.79834
67	O	-6.627426	-3.434006	-2.699268
68	N	-8.168477	-2.298737	-4.097458
69	N	-8.695947	-3.46656	-1.874793
70	C	-10.29429	-3.761114	-3.668119
71	C	-9.415081	-2.900947	-4.597366
72	C	-9.879877	-4.079323	-2.429444
73	C	-11.64784	-4.276763	-4.159244
74	C	-7.762931	-3.058173	-2.92253
75	H	-10.41972	-4.787613	-1.836674
76	H	-7.469715	-2.356799	-4.812859
77	H	-8.248674	-4.130153	-1.282746
78	H	-11.49666	-5.054164	-4.87679
79	H	-12.18551	-3.470684	-4.610037
80	H	-12.20553	-4.66027	-3.329751
81	O	-3.317725	-1.768648	2.701772
82	O	-3.137848	-5.238612	5.617666
83	N	-2.678769	-3.18922	4.533318

84	N	-4.925606	-3.677696	5.755421
85	C	-4.958533	-2.094307	3.918312
86	C	-3.530687	-2.356744	3.701544
87	C	-5.635385	-2.792896	4.799583
88	C	-5.553564	-0.976602	3.00189
89	C	-3.556802	-4.092571	5.308316
90	H	-6.697437	-2.710834	4.815613
91	H	-2.048334	-3.722466	3.954663
92	H	-5.484266	-4.490575	5.873809
93	H	-5.645983	-1.341452	1.996264
94	H	-4.89119	-0.125459	3.004235
95	H	-6.509907	-0.679951	3.362949
96	O	-1.485863	3.375218	-2.600702
97	O	-5.334385	4.596087	-4.534321
98	N	-3.695103	4.108095	-2.923512
99	N	-3.393387	5.963003	-4.722205
100	C	-1.397405	4.904078	-3.883128
101	C	-2.267689	4.055705	-3.046887
102	C	-1.930284	5.722458	-4.744966
103	C	0.152295	4.69924	-3.670631
104	C	-4.195173	4.865209	-4.081256
105	H	-1.299518	6.207612	-5.45444
106	H	-4.072553	3.173068	-2.908513
107	H	-3.684638	6.060772	-5.66354
108	H	0.462362	3.768256	-4.111208
109	H	0.370679	4.672457	-2.614884
110	H	0.692064	5.500955	-4.120862
111	O	1.560876	-3.372945	-2.685493
112	O	2.908628	-3.050632	-7.01468
113	N	2.729263	-2.627314	-4.692626
114	N	3.643491	-4.866633	-5.670178
115	C	2.633072	-4.915255	-3.471016
116	C	2.28102	-3.51202	-3.622002
117	C	3.148018	-5.582817	-4.469944
118	C	2.292598	-5.491109	-2.060617
119	C	3.077673	-3.489126	-5.846832
120	H	3.206648	-6.641464	-4.400752
121	H	2.004232	-1.978888	-4.943261
122	H	3.399977	-5.417905	-6.461071
123	H	1.22969	-5.46618	-1.912231
124	H	2.763013	-4.885721	-1.30114
125	H	2.645813	-6.49072	-1.979208
126	O	3.597331	0.98722	2.413545
127	O	6.707512	4.059927	1.325842

128	N	4.673291	3.017655	1.890551
129	N	6.675422	3.185661	3.535216
130	C	5.150172	1.310783	3.802157
131	C	4.401907	1.803381	2.624358
132	C	6.275053	1.898424	4.155548
133	C	4.531042	0.070614	4.526528
134	C	6.048268	3.44375	2.201566
135	H	6.889881	1.437058	4.894195
136	H	4.571782	2.843516	0.902234
137	H	7.662308	3.166524	3.425442
138	H	4.67987	-0.811148	3.931974
139	H	3.473739	0.227709	4.659336
140	H	4.990848	-0.061018	5.478918

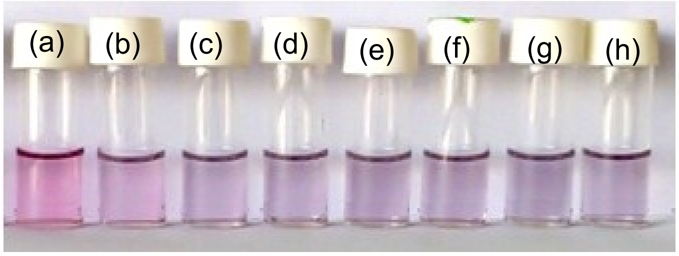

Fig. S6. Glass vial containing different dilution of hydrolyzed product of DNA standard sample and AuNPs that could be differentiated by naked eyes for qualitative determination of thymine

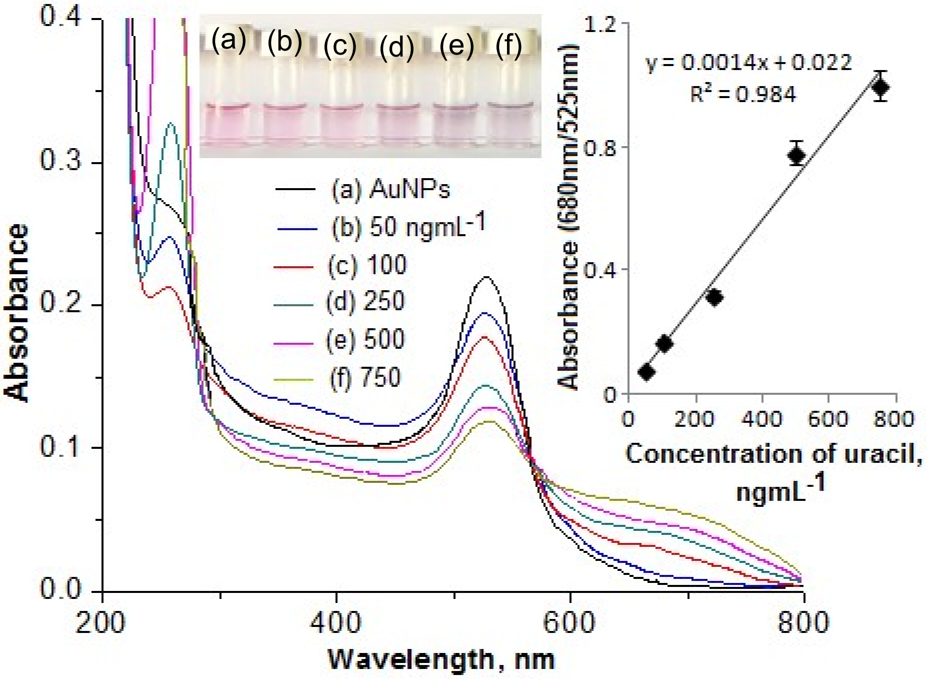

Fig. S7. Glass vial containing AuNPs along with different concentration of uracil (50, 100, 250, 500, 750 ngmL^{-1}) with their respective UV-Vis spectra and inserted graph showing the calibration curve obtained with different concentration of uracil and absorbance ratio (680 nm/525 nm)
